# Future trends of overweight and obesity in Belgium using Bayesian age-period-cohort models

**DOI:** 10.1093/eurpub/ckac131.209

**Published:** 2022-10-25

**Authors:** R De Pauw, M Claessens, V Gorasso, S Drieskens, C Faes, B Devleesschauwer

**Affiliations:** 1 Lifestyle and Chronic Diseases, Sciensano, Brussels, Belgium; 2 Rehabilitation Sciences, Ghent University, Ghent, Belgium; 3 Public Health, Ghent University, Ghent, Belgium; 4 Mathematics, University of Hasselt, Hasselt, Belgium; 5 Veterinary Sciences, Ghent University, Ghent, Belgium

## Abstract

**Background:**

Considering the current overweight and obesity epidemic and its associated increase in non-communicable diseases and healthcare costs, the current study aimed to project the trends in prevalence of overweight and obesity in Belgium using a Bayesian age-period-cohort (APC) model to support policy planning.

**Methods:**

Height and weight of 58,369 adults aged 18+ years, collected in six consecutive cross-sectional health interview surveys between 1997 and 2018, were evaluated. Criteria used for overweight and obesity were defined as body mass index (BMI) ≥ 25, and BMI ≥ 30. A Bayesian APC model was applied to evaluate past trends and associated socio-demographic risk factors, and to forecast trends to 2019-2029. All analyses were performed based on integrated nested Laplace approximation (INLA) and took the complex survey design into account.

**Results:**

The prevalence of overweight and obesity has increased between 1997 and 2018. If current trends continue, it is likely to that a further increase in the prevalence of overweight and obesity in the population will be seen by 2029 with a probability of growth of 51.2% and 73.3%, respectively. Forecasts indicated a potential prevalence of 50.1% [16.2%; 84.4%] in 2029 for overweight, and 21.4% [9.0%; 43.4%] for obesity. Among survey participants, middle-aged men with no higher education and a middle income showed the highest risk of overweight and obesity.

**Conclusions:**

We projected an alarming increase in the prevalence of overweight and obesity. A decrease in cases seems very unlikely. There is an urgent need to target younger age groups for prevention and implementation of public educational programs to limit the increasing trend in overweight and obesity.

**Key messages:**

• The occurence of obesity is likely to increase in the following 10 years.

• Projection of trends can serve as a useful tool for policy planning on the mid- and longer term.

